# Placental chorionic plate-derived mesenchymal stem cells ameliorate severe acute pancreatitis by regulating macrophage polarization via secreting TSG-6

**DOI:** 10.1186/s13287-021-02411-9

**Published:** 2021-06-10

**Authors:** Qilin Huang, Xiumei Cheng, Chen Luo, Shuxu Yang, Shuai Li, Bing Wang, Xiaohui Yuan, Yi Yang, Yi Wen, Ruohong Liu, Lijun Tang, Hongyu Sun

**Affiliations:** 1 Department of General Surgery & Pancreatic Injury and Repair Key Laboratory of Sichuan Province, The General Hospital of Western Theater Command, Chengdu, 610083 China; 2 Tianjin Medical University, Tianjin, 300070 China; 3 XinDu Hospital of Traditional Chinese Medicine & Chengdu 2nd Hospital of Traditional Chinese Medicine, Chengdu, 610500 China; 4 Division of Hepatobiliary Pancreatic Surgery, Panzhihua Central Hospital, Sichuan Province, Panzhihua, 617017 China; 5 Laboratory of Basic Medicine, The General Hospital of Western Theater Command, Chengdu, 610031 China

**Keywords:** Mesenchymal stem cells, Placenta, Severe acute pancreatitis, Macrophage polarization, TSG-6

## Abstract

**Background:**

Mesenchymal stem cells (MSCs) hold promising potential to treat systemic inflammatory diseases including severe acute pancreatitis (SAP). In our previous study, placental chorionic plate-derived MSCs (CP-MSCs) were found to possess superior immunoregulatory capability. However, the therapeutic efficacy of CP-MSCs on SAP and their underlying mechanism remain unclear.

**Methods:**

The survival and colonization of exogenous CP-MSCs were observed by bioluminescence imaging and CM-Dil labeling in rodent animal models of SAP. The therapeutic efficacy of CP-MSCs on SAP rats was evaluated by pathology scores, the levels of pancreatitis biomarkers as well as the levels of inflammatory factors in the pancreas and serum. The potential protective mechanism of CP-MSCs in SAP rats was explored by selectively depleting M1 or M2 phenotype macrophages and knocking down the expression of TSG-6.

**Results:**

Exogenous CP-MSCs could survive and colonize in the injured tissue of SAP such as the lung, pancreas, intestine, and liver. Meanwhile, we found that CP-MSCs alleviated pancreatic injury and systemic inflammation by inducing macrophages to polarize from M1 to M2 in SAP rats. Furthermore, our data suggested that CP-MSCs induced M2 polarization of macrophages by secreting TSG-6, and TSG-6 played a vital role in alleviating pancreatic injury and systemic inflammation in SAP rats. Notably, we found that a high inflammation environment could stimulate CP-MSCs to secrete TSG-6.

**Conclusion:**

Exogenous CP-MSCs tended to colonize in the injured tissue and reduced pancreatic injury and systemic inflammation in SAP rats through inducing M2 polarization of macrophages by secreting TSG-6. Our study provides a new treatment strategy for SAP and initially explains the potential protective mechanism of CP-MSCs on SAP rats.

**Supplementary Information:**

The online version contains supplementary material available at 10.1186/s13287-021-02411-9.

## Introduction

Severe acute pancreatitis (SAP) is a deadly inflammatory disease caused by local pancreatic lesions, and excessive hyperinflammation caused by immune imbalance is an important cause of systemic inflammatory response syndrome (SIRS) and secondary organ dysfunction [1, 2]. Despite intensive care treatment of SAP has improved significantly during the past few decades, the therapeutic efficacy of SAP remains unsatisfactory, with severe complications and a high mortality rate [3]. Until now, the clinical treatment strategy of SAP is still mainly based on symptomatic supportive treatment and anti-inflammatory treatment, but these treatment strategies cannot effectively correct immune imbalance that leads to excessive hyperinflammation. Therefore, it is urgent to seek for a new therapeutic strategy to re-shape the body’s immune balance in SAP.

Mesenchymal stem cells (MSCs) retain promising potential in the treatment of various inflammatory and immune diseases due to their remarkable anti-inflammatory and immunoregulatory capabilities [4–7]. However, numerous studies have demonstrated that MSCs derived from different tissues have some unique biological characteristics [8–11]. Most thrilling of all, some studies have confirmed that placental-derived MSCs (P-MSCs) not only have the advantages of rich tissue sources, easy noninvasive access, and few ethical restrictions, but also possess stronger immunoregulation and proliferation capacity. For instance, Talwadekar et al. found that P-MSCs were superior in terms of their expansion ability and immunoregulatory properties to that of umbilical cord-derived MSCs (UC-MSCs) [12]. In our prior research, we isolated and expanded three kinds of P-MSCs from different parts of the placenta, including chorionic plate-derived MSCs (CP-MSCs), chorionic villi-derived MSCs (CV-MSCs), and decidua-derived MSCs (D-MSCs), and found that CP-MSCs had stronger proliferation and migration ability than other P-MSCs and UC-MSCs [13]. Excitingly, we found that CP-MSCs expressed CD106 higher than the other three MSCs and showed stronger ability in regulating macrophage polarization from M1 to M2 [13]. Consistent with this, a study showed that CD106^**+**^ MSCs possessed stronger proliferation and immunoregulation capabilities than CD106^−^ MSCs [14]. Considering that a superior source of MSCs is crucial for cell therapy, we thus chose CP-MSCs for the treatment of SAP.

During SAP, immune imbalance triggers inflammatory cascades that lead to SIRS, multiple organ dysfunction, and even death. As a critical participator in the immune system, macrophages play a vital role in the occurrence, development, and evolution of SAP [15–17]. Intriguingly, macrophages possess strong plasticity and change their functional phenotype dependent on the local microenvironment. Some studies have confirmed that the transformation of macrophages from M1 phenotype to M2 phenotype could reduce tissue damage in various inflammatory diseases [18–20]. For instance, human bone marrow-derived MSCs (BM-MSCs) alleviate lung injury by inducing M2 polarization of macrophages in acute respiratory distress syndrome [21]. Furthermore, our group previously demonstrated that regulating the M2 polarization of peritoneal macrophage through abdominal paracentesis drainage could ameliorate systemic inflammation and pancreatic injury in SAP rats [22]. Therefore, inducing the M2 polarization of macrophages might help prevent the progression of SAP. In the present study, we transplanted exogenous CP-MSCs into rodent animal models of SAP and systematically evaluated the protective effects of CP-MSCs on SAP rats; meanwhile, we explored the regulation and potential mechanism of CP-MSCs on macrophage polarization.

## Materials and methods

### Establishment of the SAP model

Healthy wild-type male Sprague Dawley (SD) rats weighing 200~220 g were purchased from Chengdu Dossy Experimental Animal Co., Ltd. (Chengdu, China) and fed in a suitable environment with 25°C and 12 h dark/light cycle, given free access to water and food. Experimental procedures were approved by the Institutional Animal Care and Use Committee at the General Hospital of Western Theater Command and carried out in accordance with the established International Guiding Principles for Animal Research. The rats were fasted for 12 h before all surgical procedures. All experimental animals were anesthetized with isoflurane (RWD Life Science, Shenzhen, China) during the operation. The SAP models were induced by retrograde injection of 4% sodium taurocholate (TCA, 1 ml/kg body weight, Sigma, USA) into the common biliopancreatic duct as previously described [23].

### Isolation, expansion, and identification of CP-MSCs

CP-MSCs derived from the human placental chorionic plate and were cultured in MSC serum-free media (Yocon, China). The specific experimental methods of CP-MSC isolation, expansion, and identification are detailed in our previous research [13]. Immunophenotypic analysis and osteogenic and adipogenic differentiation experiments confirmed that CP-MSCs isolated from human placental chorionic plate MSCs meet the criteria of MSCs proposed by the International Society for Cellular Therapies (Fig. S1).

### Bioluminescence imaging

First, CP-MSCs were infected with lentivirus carrying a luciferase gene, and then CP-MSCs stably expressing luciferase were selected. Kunming mice were anesthetized, and 4% sodium taurocholate was injected through the pancreaticobiliary duct to prepare the SAP model. At 6 h after the operation, 1 × 10^6^ CP-MSCs expressing luciferase were infused through the tail vein. Observe the survival and distribution of CP-MSCs in SAP mice at 1 h, 24 h, 72 h, 5 days, and 7 days after exogenous CP-MSC transplantation. D-Luciferin (150 mg/kg body weight) was administered i.p. to each mouse 10 min prior to imaging. Mice were then placed in an In Vivo Imaging System (IVIS) and the photons/second emitted from the tissues were quantified using Living Image software v3.2 (Caliper Life Sciences, Alameda, CA).

### CP-MSCs in vivo tracking

Add 5μg Cell Tracker™ CM-DiI Dye (Invitrogen, USA) to 4 × 10^6^ CP-MSCs, incubate at 37°C for 5 min, and then incubate at 4°C for 15 min. After the incubation, wash twice with 1 × PBS buffer to remove unbound CM-Dil Dye, then resuspend the cell pellet in PBS buffer, and adjust the cell concentration to 2 × 10^6^ cells/ml. Ten SD rats were randomly divided into 2 groups: control group and SAP group (5 per group). CP-MSCs labeled with CM-Dil (1 × 10^6^ cells/100g) were transplanted into rats via the tail vein at 6 h and 30 h after the operation. All rats were sacrificed 72 h after the first CP-MSC transplantation; the lung, heart, liver, pancreas, spleen, kidney, duodenum, and colon were collected and then fixed in 4% paraformaldehyde for 24 h and dehydrated in 30% sucrose solution. Subsequently, the tissues were embedded in Tissue Freezing Medium and cut into 8-μm-thick sections. The slides were washed with PBS and stained with DAPI to visualize the nuclei. The distribution of CP-MSCs in different organs was observed under a fluorescence microscope.

### CP-MSC transplantation in SAP rats

Thirty-two SD rats were randomly divided into 4 groups: control group, control + CP-MSC group, SAP group, and SAP + CP-MSC group (8 per group). In the CP-MSC intervention group, CP-MSCs (1 × 10^6^ cells/100g) were delivered through tail vein 6 h and 30 h after the operation. All rats were sacrificed 72 h after the first CP-MSC transplantation, serum, liver, and pancreas tissues were collected.

### Histopathology

Pancreas samples were fixed in 10% buffered formaldehyde, embedded in paraffin, and sectioned. The 4-μm-thick deparaffinized sections were stained with H&E for routine histology. According to the scoring criteria reported by Schmidt et al. [24], the degree of pancreatic edema, acinar cell necrosis, hemorrhage, and inflammatory infiltrate were scored. Five different fields were randomly observed under the microscope each slide.

### Cell Counting Kit-8 (CCK-8) assay

The CCK-8 assay was used to detect the effect of CM-Dil on the proliferation of CP-MSCs. The detailed operating steps were seen in *supplementary materials*.

### Enzyme-linked immunosorbent assay (ELISA)

Inflammatory factors (IL-1β, IL-6, TNF-α, TGF-β, IL-4, and IL-10), amylase, and lipase in rat serum were detected by ELISA kits (Shanghai Jianglai Biotech, China). In addition, the human tumor necrosis factor-α-induced gene/protein 6 (TSG-6) ELISA kit (Shanghai Jianglai Biotech, China) was used to detect the concentration of TSG-6 in the culture supernatant of CP-MSCs. Detailed operating steps were according to the manufacturer’s instructions.

### Detection of myeloperoxidase (MPO) activity in pancreatic tissue

Accurately weigh pancreatic tissue of the same quality, then grind it into a homogenate, and follow the manufacturer’s instructions in the kit to detect MPO activity in the pancreatic tissue of each group.

### Real-time quantitative PCR (RT-qPCR)

Total RNA was extracted using Trizol reagent (Invitrogen Inc., USA), according to the products’ instructions. The RNA was quantified by measuring the absorbance at 260nm and 280nm using a spectrophotometer (NanoDrop Technologies, USA). RT-qPCR was performed with a CFX96 Real-Time PCR Detection System (Bio-Rad, USA) using one-step SYBR PrimeScript RT-PCR Kit (TaKaRa, Japan). The sequences of primers are listed in supplementary Table S1.

### Immunofluorescence staining

Immunofluorescence staining is used to detect the polarizing phenotype of macrophages in pancreas and liver tissues, and the detailed method is described in *supplementary materials*.

### Preparation and polarization induction of bone marrow-derived macrophages

Bone marrow (BM)-derived macrophages were isolated from SD rats by flushing the BM with DMEM (Hyclone, USA) as previously described [25, 26]. Bone marrow macrophage induction medium was used to induce differentiation of precursor cells into macrophages. After 7–10 days in culture, nonadherent cells were removed and adherent cells were ready for experiment. Macrophages were induced with an M1 or M2 polarization induction medium for 24 h and then collected for subsequent experiments.

Bone marrow macrophage induction medium: DMEM + 10% FBS (Gibico, USA), 10 ng/ml M-CSF (Peprotech, USA).

Macrophage M1 polarization induction medium: DMEM + 10% FBS, 100 ng/ml LPS (Sigma, USA), 50 ng/ml IFN-γ (Peprotech, USA).

Macrophage M2 polarization induction medium: DMEM + 10% FBS, 10 ng/ml IL-10 (Peprotech, USA), 10 ng/ml IL-13 (Peprotech, USA).

### Selective depletion of M1 or M2 macrophages

#### In vitro experiment

Unpolarized-induced macrophages (M0), M1 polarization-induced macrophages (M1), and M2 polarization-induced macrophages (M2) were inoculated into six-well plates at 1 × 10^6^ cells/well. After attachment of macrophages, GdCl3 (100 μM, Sigma, USA) or mannosylated clodronate-encapsulated liposomes (MCLs, Encapsula Nano Sciences, USA) were added to the macrophage medium. The volume ratio of MCLs to the culture medium is 1:100. Macrophages were collected for apoptosis analysis after 48 h of intervention.

#### In vivo experiment

Thirty-six SD rats were randomly divided into 6 groups: SAP group, SAP + CP-MSC group, SAP + GdCl3 group, SAP + GdCl3 + CP-MSC group, SAP + MCLs group, and SAP + MCLs + CP-MSC group (6 per group). In the GdCl3 intervention group, the GdCl3 solution (0.5%, 20 mg/kg) was infused via the tail vein immediately after the operation. In the MCL intervention group, 1 ml of MCL solution was infused via the tail vein immediately after the operation. In the CP-MSC intervention group, CP-MSCs (1× 10^6^ cells/100g) were delivered through the tail vein 6 h and 30 h after the operation. All rats were sacrificed 72 h after the first CP-MSC transplantation, and serum, liver, and pancreas tissues were collected.

### Flow cytometric analysis of macrophage apoptosis

Macrophage apoptosis was detected using Annexin V-FITC Apoptosis Detection Kit (Beijing Solarbio Science & Technology Co., Ltd., China), and the detailed operating steps are described in *supplementary materials*.

### TSG-6 shRNA transfection

CP-MSCs were transfected with TSG-6-specific or nonspecific control short hairpin A (shRNA, Shanghai Genechem Co., Ltd., China) using transfection reagent in shRNA transfection media according to the manufacturer’s protocol. Puromycin (Sigma, USA) was employed to select stable knockdown cells for at least three passages.

### CP-MSC intervened with SAP rat serum

To simulate the microenvironment of CP-MSCs in SAP rats, CP-MSCs were cultured in MSC serum-free media containing 0%, 25%, and 50% SAP rat serum. After 12 h of cultivation, CP-MSCs were collected for RT-qPCR assay.

### Macrophages and CP-MSC noncontact co-culture

To explore the effects of different polarized phenotype macrophages on CP-MSCs, CP-MSCs and macrophages (M1 or M2 macrophages) were co-cultured at 2:5 through transwell chamber (0.4-μm pore size; Corning, USA), where CP-MSCs and macrophages were located in the lower and upper compartment of the chamber respectively. After 24 h of co-cultivation, CP-MSCs were collected for RT-qPCR assay.

To explore the mechanism of CP-MSCs regulating macrophage polarization, M1 macrophages were inoculated in six-well plates, and then CP-MSCs (TSG-6 shRNA) or CP-MSCs (scr shRNA) were inoculated in the upper layer of the transwell chamber (0.4-μm pore size; Corning, USA); the ratio of macrophages to CP-MSCs is 5:1. After 24 h of co-cultivation, the polarization phenotype of macrophages was detected by flow cytometry and RT-qPCR.

### Flow cytometry analysis

The polarized phenotype of macrophages was analyzed using the following antibodies: FITC-conjugated CD163 (Bio-Rad, USA), PE-conjugated CD86 (BD Biosciences, USA), and Alexa-Flour647-conjugated CD68 (Bio-Rad, USA). Nonspecific isotype-matched antibodies served as controls. The cells were analyzed using a flow cytometry instrument (BD CantoII, USA) and the data were analyzed using FlowJo V10.

### Statistical analysis

Statistics as well as graphical representations were performed using GraphPad Prism™ 7.0 (GraphPad Software Inc., USA). All data are expressed as the means ± SEM. Comparisons between two groups were performed using Student’s t-test. Comparisons between more than two groups were analyzed by a one-way ANOVA test. Results were considered statistically significant when P < 0.05.

## Results

### Survival and distribution of exogenous CP-MSCs in rodent animal models of SAP

**Fig. 1:**
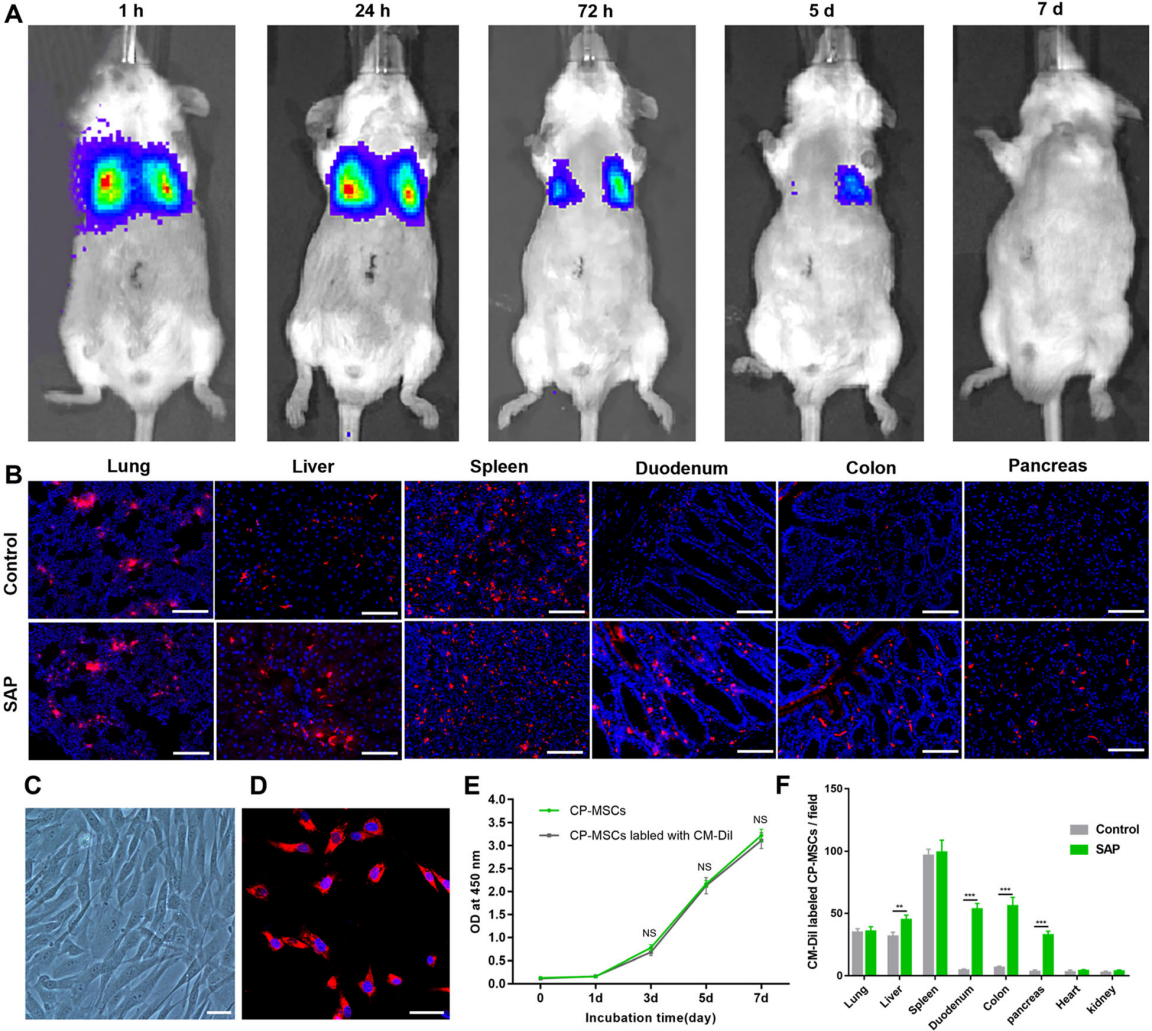
Survival and distribution of exogenous CP-MSCs in rodent animal models of SAP. **A** The bioluminescence imaging was used to monitor the survival status of exogenous CP-MSCs in SAP at 7-day intervals continuously. **B** CP-MSCs were labeled with CM-Dil to track the distribution of CP-MSCs in SAP rats; all rats were sacrificed 72 h after the first CP-MSC transplantation; and the lung, heart, liver, pancreas, spleen, kidney, duodenum, and colon were collected, then observed the distribution of CP-MSCs in different tissues under a fluorescence microscope. Scale bars, 200 μm. **C** CP-MSCs without CM-Dil labeling. Scale bars, 50 μm. **D** CP-MSCs labeled with CM-Dil were inoculated in a culture flask and placed in a cell culture incubator, and CM-Dil does not affect the proliferation of CP-MSCs. Scale bars, 50 μm. **E** The CCK-8 assay was used to evaluate the effect of CM-Dil on the proliferation of CP-MSCs. Data are represented as mean ± SEM (n = 6). **F** Distribution was assessed from the lung, liver, spleen, duodenum, colon, pancreas, heart, and kidney sections after CM-Dil-labeled with CP-MSC injection in rats with or without SAP. The numbers of CM-Dil-labeled CP-MSCs represent the mean ± SEM of at least 5 fields (n = 5). Significance is indicated as follows: *p < 0.05, **p < 0.01, ***p < 0.001; NS, no significant

The survival of exogenous CP-MSCs in the hyperinflammatory environment of SAP is the basis for therapeutic effects. Therefore, the bioluminescence imaging was used to monitor the survival status of CP-MSCs in SAP mice. The results showed that the number of surviving CP-MSCs decreased significantly when CP-MSCs were transplanted into SAP for 72 h, while no fluorescent signal could be detected in vivo after CP-MSCs were transplanted for 7 days (Fig. 1A). As CP-MSC migration and recruitment are crucial to the success of CP-MSC-mediated immune regulation, we determined whether exogenous CP-MSCs may respond to signals of cellular damage to the sites of injury after SAP. To track the distribution and colonization of CP-MSCs in SAP rats, CP-MSCs were labeled with CM-Dil and adoptively transferred into SAP rats via tail vein. Indeed, we found that there were more CP-MSCs (red) colonized in the liver, pancreas, duodenum, and colon of the SAP group compared to the control group (Fig. 1B, F). Moreover, to observe whether CM-Dil affects the proliferation of CP-MSCs, CP-MSCs were labeled with CM-Dil, inoculated in the culture flask, and placed in a cell culture incubator. We found that CM-Dil had no significant effects on the proliferation and morphology of CP-MSCs (Fig. 1C, D). Meantime, we confirmed that CM-Dil had no significant effects on the proliferation of CP-MSCs by CCK-8 assay (Fig. 1E).

### CP-MSCs could alleviate pancreatic injury and systemic inflammatory

**Fig. 2:**
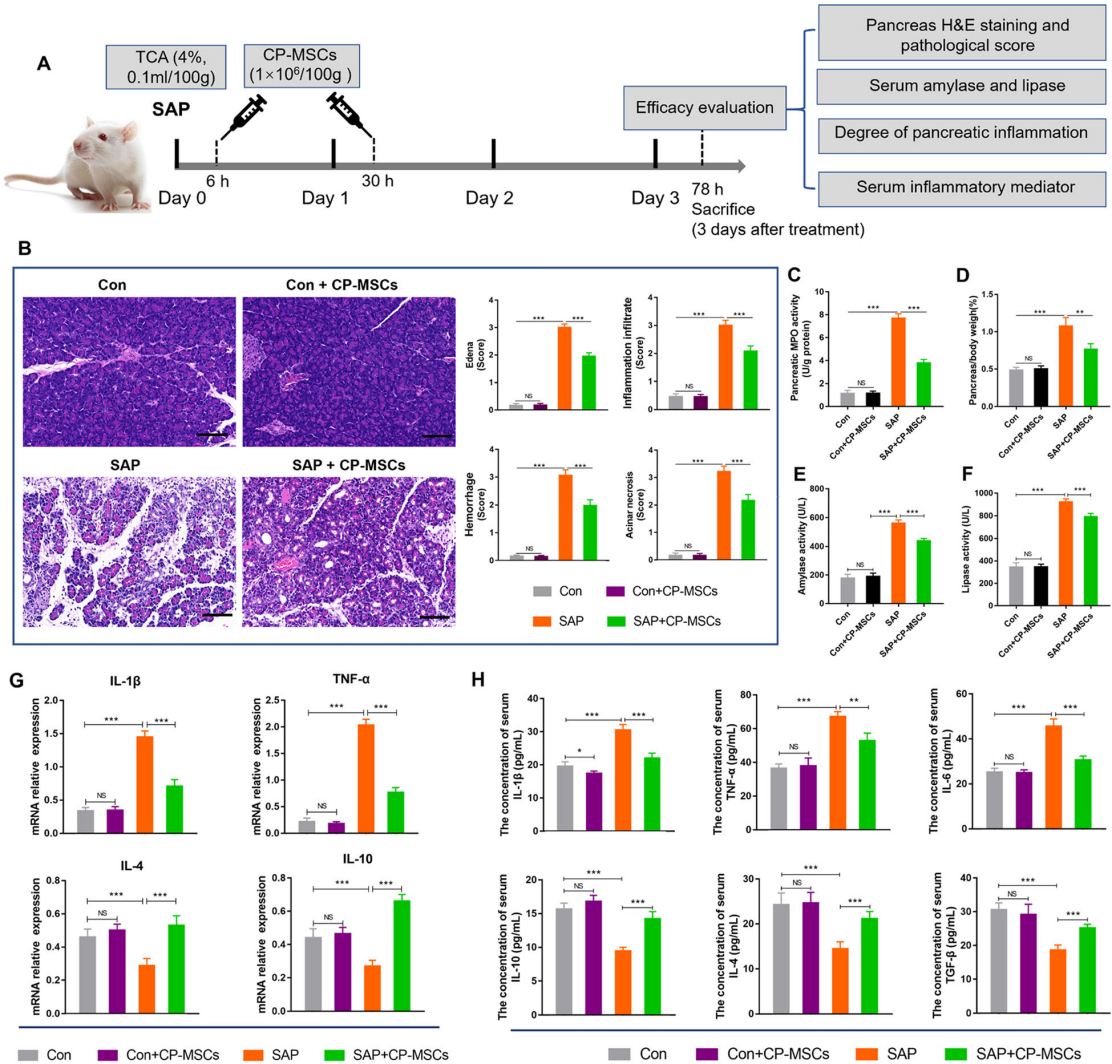
CP-MSCs reduced pancreatic injury and systemic inflammatory in SAP rats. **A** Schematic diagram of exogenous CP-MSC transplantation in SAP rats. TCA, sodium taurocholate. **B** H&E staining of pancreas tissue. Scale bars, 50 μm. Histopathological scores for the pancreas, including edema, inflammation infiltrate, acinar necrosis, and hemorrhage. **C** Pancreatic myeloperoxidase (MPO) activity. MPO is unique to mature neutrophils, so pancreatic MPO activity can be used to reflect the infiltration of neutrophils in the pancreas. **D** Pancreas/body-weight ratio is used to quantify pancreatic edema. **E**, **F** Serum amylase and lipase activity. **G** The mRNA expression levels of pro-inflammatory cytokines (IL-1β and TNF-α) and anti-inflammatory cytokines (IL-4 and IL-10) in the pancreatic tissue. **H** The concentrations of pro-inflammatory cytokines (IL-1β, TNF-α, and IL-6) and anti-inflammatory cytokines (IL-10, IL-4, and TGF-β) in serum. All graphs show mean ± SEM (n = 8). Significance is indicated as follows: *p < 0.05; **p < 0.01; ***p < 0.001; NS, no significant

To assess the therapeutic efficacy of CP-MSCs on pancreatic injury in SAP rats, we first performed pancreatic histopathology scores and measured the activity of amylase and lipase in serum and pancreatic MPO activity (Fig. 2A). Histologically, the SAP group showed obvious morphological damage, such as edema, inflammation infiltrate, acinar necrosis, and hemorrhage, whereas the pancreatic tissue damage was significantly reduced in the SAP + CP-MSC group (Fig. 2B). In addition, compared with the SAP group, pancreatic MPO activity, pancreas/body-weight ratio, and serum amylase and lipase activity were significantly reduced in the SAP + CP-MSC group (Fig. 2C–F). Meanwhile, RT-qPCR results of inflammatory factor mRNA in pancreatic tissue showed that CP-MSCs could significantly reduce the expression levels of pro-inflammatory factors IL-1β and TNF-α and increase the expression levels of anti-inflammatory factors IL-4 and IL-10 (Fig. 2G). Finally, we explored the effects of CP-MSCs on the systemic inflammatory in SAP rats. ELISA experimental data showed that compared with the SAP group, in the SAP + CP-MSC group, the serum concentrations of pro-inflammatory cytokines (such as IL-1β, TNF-α, and IL-6) significantly decreased, while the concentrations of anti-inflammatory cytokines (such as IL-10, IL-4, and TGF-β) increased significantly (Fig. 2H). The above data fully illustrated that exogenous CP-MSCs could reduce pancreatic injury and systemic inflammation in SAP rats.

### CP-MSCs induced M2 polarization of macrophages

**Fig. 3:**
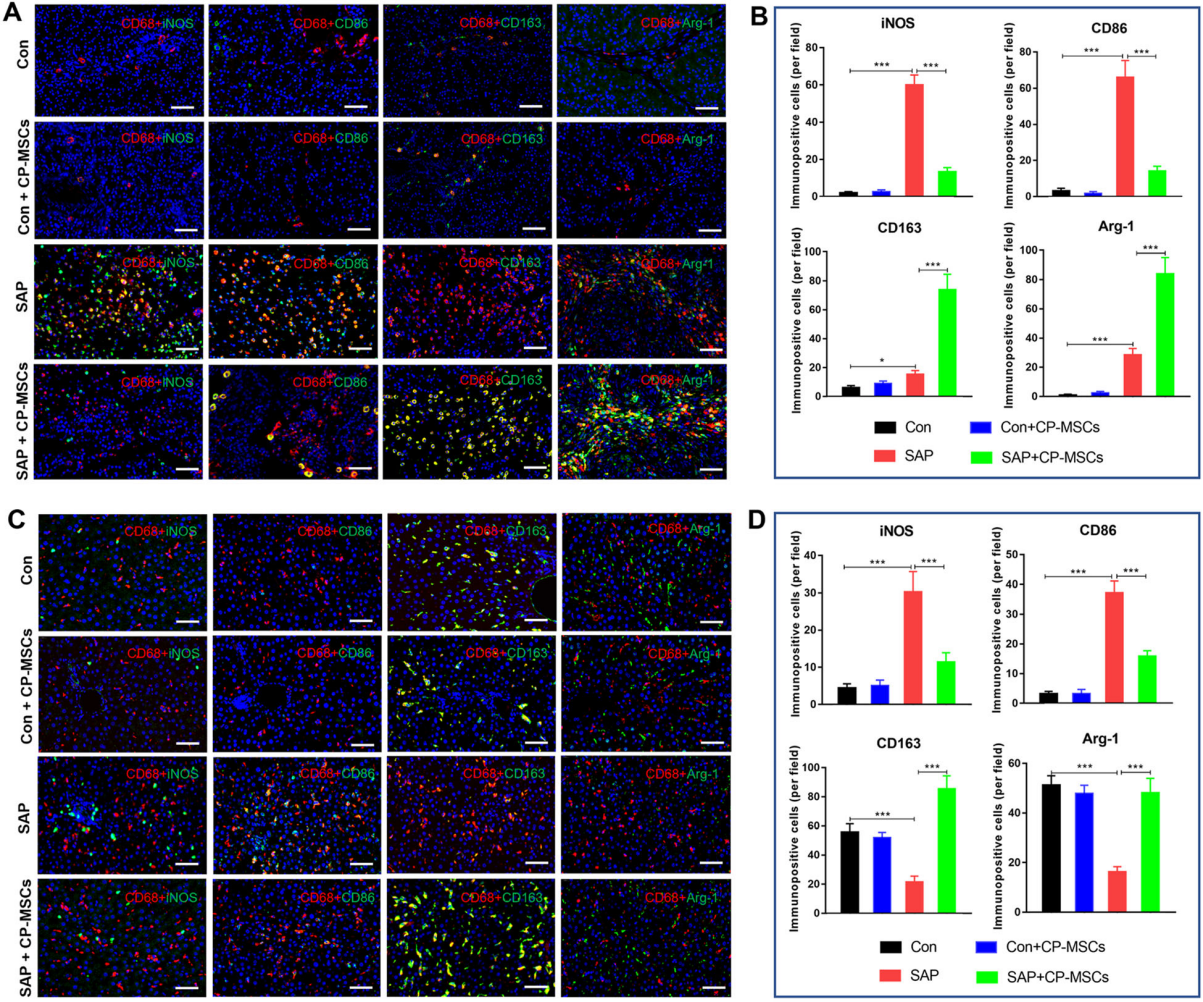
CP-MSCs induced M2 polarization of macrophages in SAP rats. To observe the effect of CP-MSCs on macrophage polarization in vivo, all rats were sacrificed 72 h after the first CP-MSC transplantation, liver and pancreas tissues were collected, and then the polarization phenotype of macrophages was detected by immunofluorescence staining. **A** Representative immunofluorescence staining of the polarized phenotype of macrophages in the pancreas tissue. CD68^+^/iNOS^+^ and CD68^+^/CD86^+^ are considered as M1 macrophages, and CD68^+^/CD163^+^ and CD68^+^/Arg-1^+^ are considered as M2 macrophages. Scale bars, 50 μm. **B** Percentage of M1 macrophages (iNOS^+^, CD86^+^) and M2 macrophages (CD163^+^, Arg-1^+^) per field in the pancreas. **C** Representative immunofluorescence staining of the polarized phenotype of macrophages in the liver tissue. Scale bars, 50 μm. **D** Percentage of M1 macrophages (iNOS^+^, CD86^+^) and M2 macrophages (CD163^+^, Arg-1^+^) per field in the liver tissue. The numbers of immunopositive cells represent the mean ± SEM of at least 5 fields (n = 5). Significance is indicated as follows: *p < 0.05; **p < 0.01; ***p < 0.001

Increasing evidence has shown that M2 polarization of macrophages could mitigate tissue inflammatory and damage [21, 27, 28], so exploring the regulation of CP-MSCs on macrophage polarization would provide strong evidence for revealing its potential therapeutic mechanism in SAP rats. The results of immunofluorescence staining of the polarized phenotype of macrophages showed that there were only a few macrophages (CD68^+^) in the pancreas tissues of the Con group and Con + CP-MSC group, and these macrophages were mainly located in the lobular space of the pancreas, beside blood vessels or bile ducts (Fig. 3A). Compared with the Con group, there were a large number of macrophages in the pancreas and liver tissues of the SAP group, and these macrophages highly expressed CD86 and iNOS, while very few expressed CD163 and Arg-1 (Fig. 3A–D). Compared with the SAP group, the number of macrophages in the pancreatic tissue of the SAP + CP-MSC group was slightly reduced, and the expression of CD86 and iNOS in the pancreas and liver tissue macrophages were significantly decreased, while the expression of CD163 and Arg-1 were significantly increased (Fig. 3A–D). Based on these results, we inferred that CP-MSCs could induce the polarization of macrophages from M1 to M2 in the pancreas and liver tissues of SAP rats.

### CP-MSCs mitigated pancreatic injury and systemic inflammatory mainly by inducing M2 polarization of macrophages

**Fig. 4:**
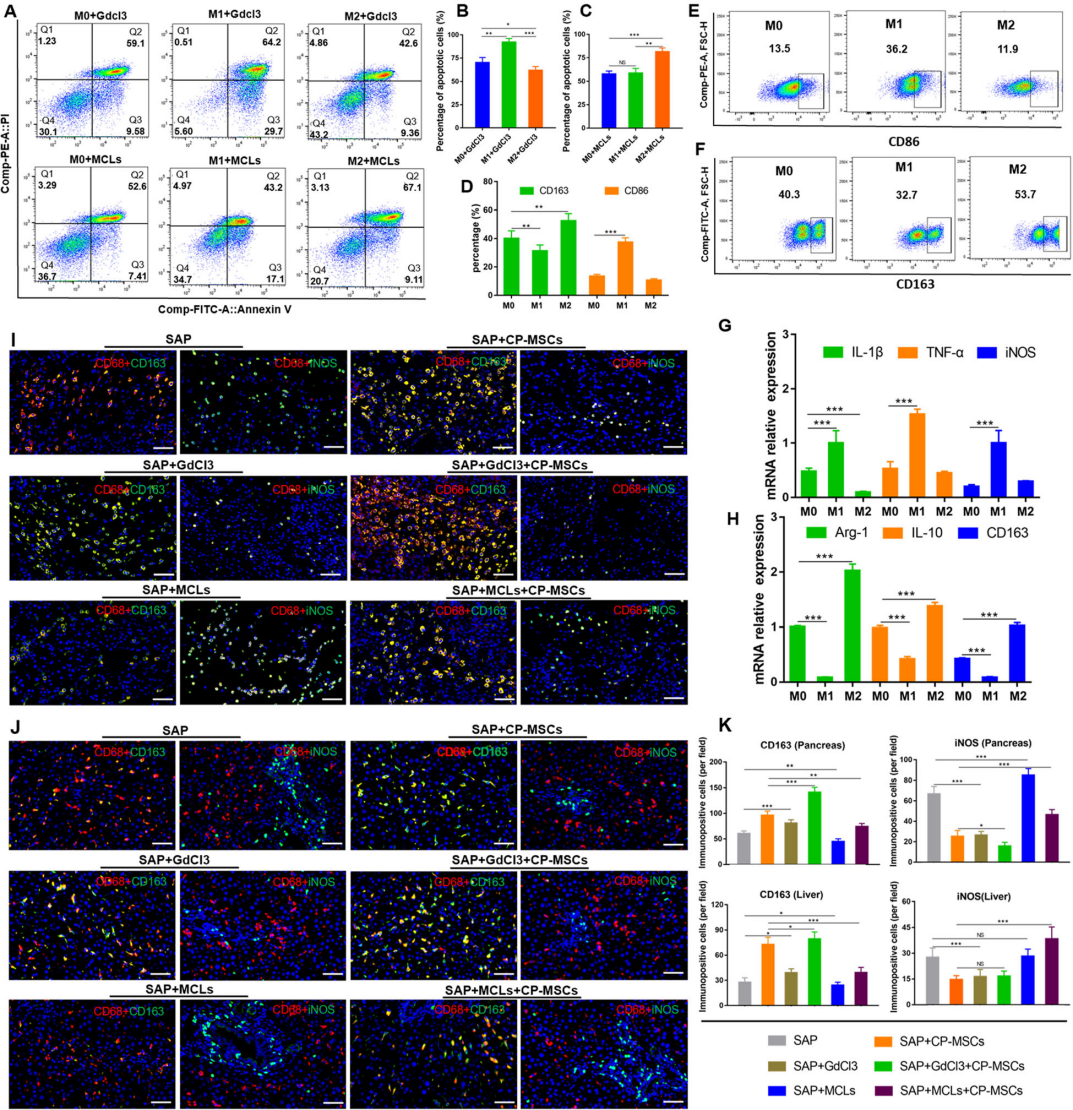
GdCl3 and MCLs selectively induced apoptosis of M1 or M2 macrophages. To investigate the effects of GdCL3 and MCLs on macrophages with different polarization phenotypes in vitro, we isolated and cultured macrophages from the bone marrow and induced the macrophages into M1 and M2 phenotypes. **A** Flow cytometry analysis of the apoptosis of macrophages with different polarization phenotypes after 48-h intervention with gadolinium (III) chloride (GdCl3) or mannosylated clodronate-encapsulated liposomes (MCLs). **B**, **C** Quantitative results of the apoptosis rate when macrophages with different polarization phenotypes were intervened by GdCl3 or MCLs (n = 3). The total apoptotic number of macrophages is from AnV+ and Anv/PI+. To confirm that we successfully induced bone marrow cells into M1 and M2 macrophages in vitro, the polarization phenotype of macrophages was analyzed by flow cytometry (**D**) and RT-qPCR (**G**, **H**), CD163^+^ macrophages are considered as M2 macrophages, and CD86^+^ macrophages are considered as M1 macrophages. **E**, **F** Flow cytometric analysis of macrophages with different polarization phenotypes (n = 3). **G** The mRNA expression levels of M1 marker genes (IL1-β, TNF-α, and iNOS) in macrophages (n = 3). **H** The mRNA expression levels of M2 marker genes (Arg-1, IL-10, and CD163) in macrophages (n = 3). To observe the effect of CP-MSCs on macrophage polarization in vivo, all rats were sacrificed 72h after the first CP-MSC transplantation, liver and pancreas tissues were collected, and then the polarization phenotype of macrophages was detected by immunofluorescence staining. Representative immunofluorescence staining of the polarized phenotype of macrophages in the pancreas (**I**) and liver tissues (**J**). Scale bars, 50 μm. **K** Quantification of M2 macrophages (CD163^+^) and M1 macrophages (iNOS^+^) in the pancreas and liver tissue, the numbers of immunopositive cells represent the mean ± SEM of at least 5 fields (n = 5). All graphs show mean ± SEM. Significance is indicated as follows: *p < 0.05; **p < 0.01; ***p < 0.001; NS, no significant. M0 represents unpolarization-induced bone marrow macrophages; M1 represents M1 polarization-induced bone marrow macrophages; M2 represents M2 polarization-induced bone marrow macrophages

**Fig. 5:**
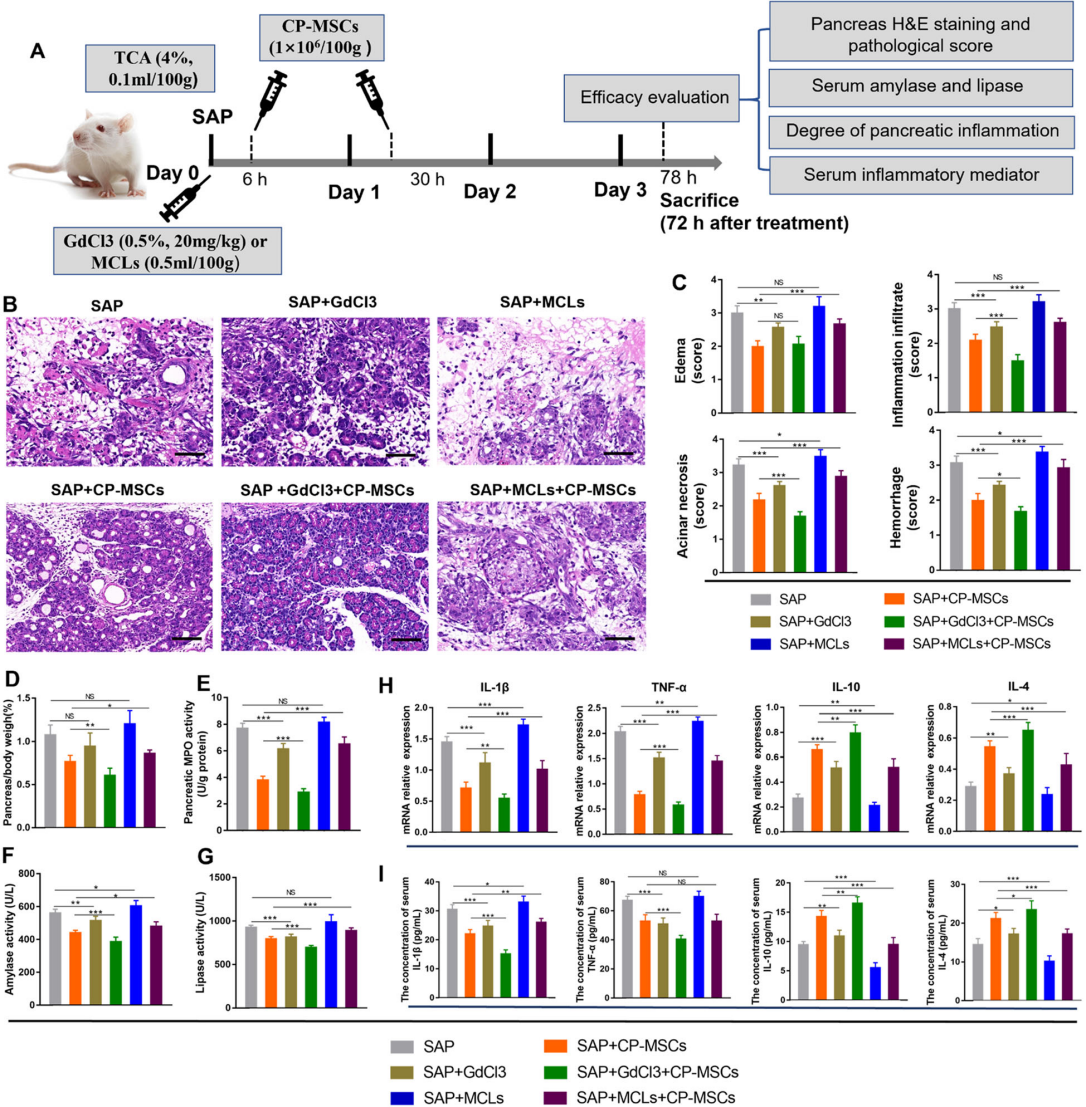
The protective effect of CP-MSCs on SAP rats is significantly weakened after the depletion of M2 macrophages. **A** Schematic diagram of exogenous CP-MSC transplantation in SAP rats. GdCl3 or MCLs were injected into the SD rats via the tail vein immediately after the operation, and CP-MSCs were injected via the tail vein at 6h and 30h after operation. The therapeutic effect of CP-MSCs on SAP rats was evaluated 72h after the first CP-MSC transplantation. **B** H&E staining of pancreas tissue. Scale bars, 50 μm. **C** Histopathological scores for the pancreas, including edema, inflammation infiltrate, acinar necrosis, and hemorrhage. **D** Pancreas/body-weight ratio. **E** Pancreatic MPO activity. Serum amylase (**F**) and lipase (**G**) activity. **H** The mRNA expression levels of pro-inflammatory factors (IL-1β and TNF-α) and anti-inflammatory factors (IL-10 and IL-4) in the pancreatic tissue. **I** The concentrations of pro-inflammatory factors (IL-1β and TNF-α) and anti-inflammatory factors (IL-4 and IL-10) in serum. All graphs show mean ± SEM (n = 6). Significance is indicated as follows: *p < 0.05; **p < 0.01; ***p < 0.001; NS, no significant

To further explore whether CP-MSCs exerted a therapeutic role by regulating the polarization of macrophages from M1 to M2 in SAP rats, we selectively deplete M1 or M2 macrophages when CP-MSCs were administered, and then observe the protective effects of CP-MSC on SAP rats. We depleted M1 macrophages by administration of GdCl3, which upon phagocytosis induces apoptosis of inflammatory macrophages (M1) via competitive inhibition of Ca2^+^ mobilization and damage to plasma membranes [29]. We depleted M2 macrophages using mannosylated clodronate liposomes (MCLs) that bind the mannose receptor which is upregulated following M2 polarization, and induce apoptosis via clodronate-mediated depletion of intracellular iron [30, 31]. First, we successfully obtained macrophages from rat bone marrow (Figure S3) and induced them into M1 and M2 macrophages (Fig. 4D–H). Next, we confirmed that GdCl3 could relatively selectively induce apoptosis of M1 macrophages (Fig. 4A, B), and MCLs could relatively selectively induce apoptosis of M2 macrophages in vitro (Fig. 4A, C). Finally, GdCl3 or MCLs were infused into SAP rats through the tail vein before CP-MSC transplantation (Fig. 5A), and then the polarization phenotype of macrophages in the liver and pancreas was detected by immunofluorescence staining. We found that when SAP rats were given GdCl3 intervention, iNOS^+^ macrophages in the pancreas and liver tissues decreased significantly, while CD163^+^ macrophages increased; when SAP rats were given MCL intervention, CD163^+^ macrophages in pancreas and liver tissues decreased significantly, while iNOS^+^ macrophages increased (Fig. 4I–K). Therefore, the above data manifested that GdCl3 selectively depleted M1 macrophages, and MCLs selectively depleted M2 macrophages in the pancreas and liver tissues of SAP rats.

Moreover, through pancreatic H&E staining and pathological scores (Fig. 5B, C), the pancreas/body-weight ratio (Fig. 5D), pancreatic MPO activity (Fig. 5E), RT-qPCR results of pancreatic inflammatory factors mRNA (Fig. 5H), and ELISA data of serum inflammatory factors (Fig. 5I), amylase (Fig. 5F), and lipase (Fig. 5G), we found that pancreatic damage and systemic inflammation were significantly reduced when M1 macrophages were depleted, and pancreatic damage and systemic inflammation were significantly worsened when M2 macrophages were depleted. Therefore, it is showed that M1 macrophages aggravated tissue inflammation and injury in SAP rats, while M2 macrophages promoted the regression of tissue inflammation and repaired the injured tissues. In addition, we found that the therapeutic efficacy of CP-MSCs was significantly enhanced when M1 macrophages were depleted, while the therapeutic efficacy of CP-MSCs was significantly inhibited when M2 macrophages were depleted in SAP rats. Therefore, it is indicated that CP-MSCs attenuated pancreatic injury and systemic inflammation mainly by inducing M2 polarization of macrophages in SAP rats.

### CP-MSCs induced M2 polarization of macrophages by secreting TSG-6

**Fig. 6:**
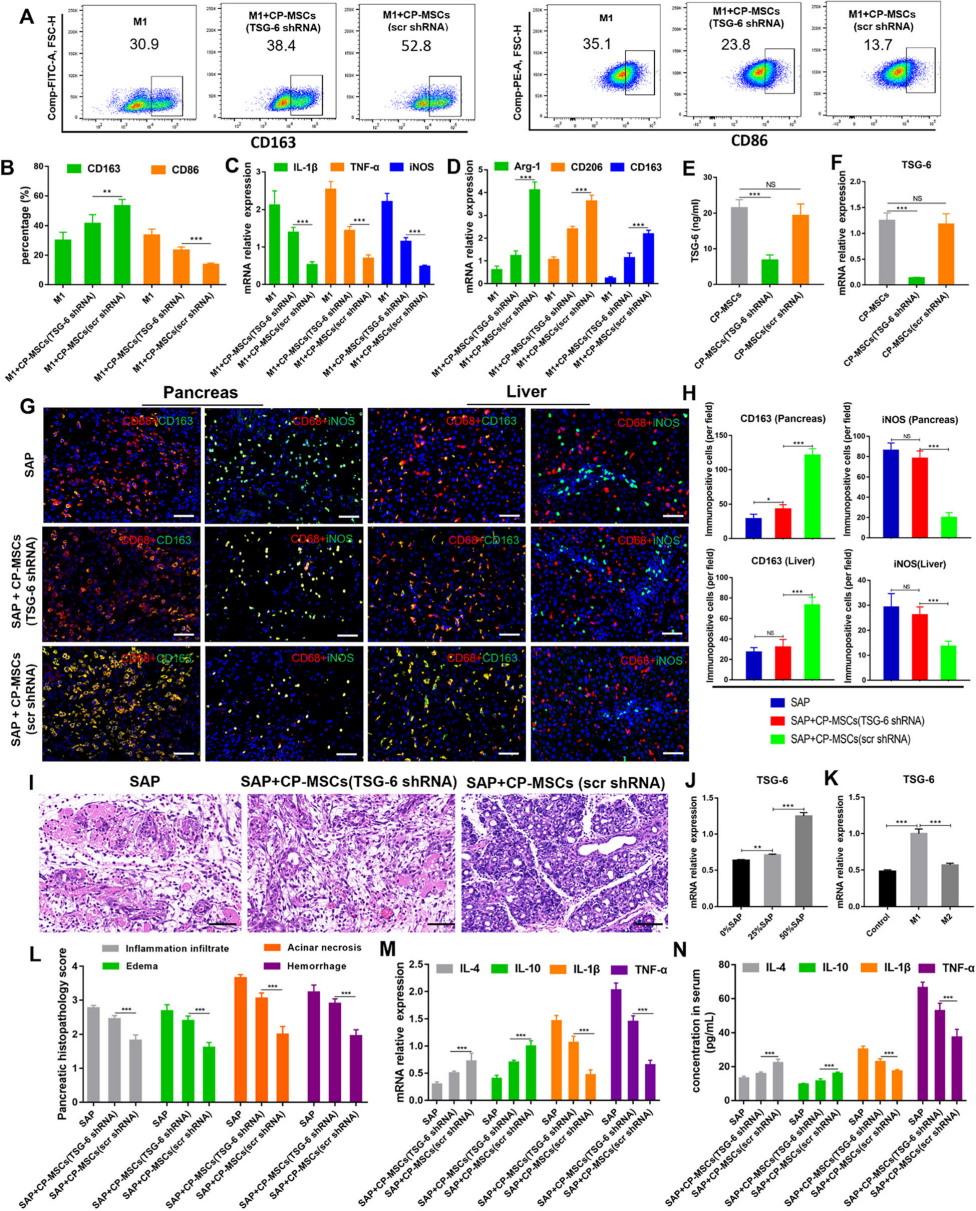
CP-MSC-derived TSG-6 played a vital role in inducing M2 polarization of macrophages and reducing pancreatic injury and systemic inflammation in SAP rats. **A** Flow cytometric analysis of the macrophage polarization phenotype after CP-MSCs (TSG-6 shRNA) or CP-MSCs (scr shRNA) co-cultured with M1 macrophages for 24 h. **B** Quantification of macrophage polarization phenotype. CD86^+^ macrophages are considered as M1 macrophages, and CD163^+^ macrophages are considered as M2 macrophages (n = 3). **C** The mRNA expression levels of M1 marker genes (IL1-β, TNF-α, and iNOS) in macrophages (n = 3). **D** The mRNA expression levels of M2 marker genes (Arg1, IL-10, and CD163) in macrophages (n = 3). TSG-6 protein (**E**) and mRNA (**F**) expression levels in CP-MSCs (TSG-6 shRNA), CP-MSCs (scr shRNA), and CP-MSCs (n = 3). **G** Representative immunofluorescence staining of the polarized phenotype of macrophages in the pancreas and liver tissue. Scale bars, 50 μm. **H** Quantification of M2 macrophages (CD163^+^) and M1 macrophages (iNOS^+^) in the pancreas and liver tissue. The numbers of immunopositive cells represent the mean ± SEM of at least 5 fields (n = 5). **I** H&E staining of pancreas tissue. Scale bars, 50 μm. **J** RT-qPCR analysis of TSG-6 expression level of CP-MSCs after intervention by different concentrations of SAP rat serum (n = 3). **K** RT-qPCR analysis of the TSG-6 expression level of CP-MSCs after CP-MSCs were co-cultured with M1 macrophages or M2 macrophages for 24 h (n = 3). **L** Histopathological scores for the pancreas showed the degree of edema, inflammation infiltrate, hemorrhage, and acinar necrosis (n = 5). **M** The mRNA expression levels of pro-inflammatory factors (IL-1β and TNF-α) and anti-inflammatory factors (IL-4 and IL-10) in the pancreas (n = 5). **N** The concentrations of pro-inflammatory factors (IL-1β and TNF-α) and anti-inflammatory factors (IL-4 and IL-10) in rat serum (n = 5). Data are presented as mean ± SEM. Significance is indicated as follows: *p < 0.05; **p < 0.01; ***p < 0.001; NS, no significant

To verify that CP-MSCs regulate the polarization of macrophages from M1 to M2 by secreting TSG-6, RNA interference technology was used to inhibit the expression of TSG-6 in CP-MSCs. By measuring the concentration of TSG-6 in the culture supernatant (Fig. 6E) and the expression level of TSG-6 gene (Fig. 6F), it was confirmed that the expression of TSG-6 of CP-MSCs was successfully inhibited. Flow cytometric analysis showed that compared with the M1 + CP-MSC (scr shRNA) group, CD163^+^ macrophages were significantly reduced and CD86^+^ macrophages were significantly increased in the M1 + CP-MSC (TSG-6 shRNA) group (Fig. 6A, B). In addition, RT-qPCR results indicated that compared with the M1 + CP-MSC (scr shRNA) group, the mRNA expression levels of M1 macrophage marker genes (IL-1β, TNF-α, and iNOS) were significantly increased, and M2 macrophage marker genes (Arg-1, IL-10, and CD163) were significantly reduced in the M1 + CP-MSC (TSG-6 shRNA) group (Fig. 6C, D). The above data manifested that TSG-6 secreted by CP-MSCs played an important role in regulating the polarization of macrophages from M1 to M2.

### CP-MSC-derived TSG-6 alleviated SAP by suppressing pancreatic and systemic inflammation

In the preceding, we have shown that CP-MSC-derived TSG-6 shifted the macrophages from a pro-inflammatory phenotype (M1) to an anti-inflammatory phenotype (M2) in vitro. Next, we explored whether CP-MSC-derived TSG-6 is involved in the switch of anti-inflammatory macrophages in SAP rats. Polarized phenotype immunofluorescence staining of macrophages showed that when the expression of CP-MSC-derived TSG-6 was suppressed, iNOS^+^ macrophages (M1) increased significantly and CD163^+^ macrophages (M2) decreased significantly in the pancreas and liver tissues of SAP rats (Fig. 6G, H).

To confirm that CP-MSCs exert a therapeutic role mainly by secreting TSG-6, CP-MSCs (TSG-6 shRNA) were transplanted into SAP rats to evaluate the therapeutic effect. Pancreatic HE staining and pathology scores (Fig. 6I, L), RT-qPCR results of inflammatory factors in pancreatic tissues (Fig. 6M), and ELISA data of serum inflammatory factors (Fig. 6N) indicated that when the expression of CP-MSC-derived TSG-6 was suppressed, the therapeutic effect of CP-MSCs is significantly weakened. Therefore, it showed that TSG-6 secreted by CP-MSCs played a vital role in reducing pancreatic injury and systemic inflammation.

Moreover, we found that when SAP rat serum was used to simulate the hyperinflammatory environment on CP-MSCs in SAP rats, SAP rat serum significantly stimulated CP-MSCs to express TSG-6 in a dose-dependent manner (Fig. 6J). In addition, we found that when CP-MSCs were co-cultured with macrophages, M1 macrophages stimulated CP-MSCs to express more TSG-6, while M2 macrophages did not significantly affect the expression of TSG-6 in CP-MSCs (Fig. 6K). Therefore, the above results indicated that a hyperinflammatory environment could stimulate CP-MSCs to express more TSG-6.

## Discussion

In the present study, we provided the first evidence that exogenous CP-MSCs attenuated SAP by inducing macrophage polarization from M1 to M2 via secreting TSG-6. The important findings of this study are as follows: (i) Exogenous CP-MSCs can survive in the hyperinflammatory environment of SAP and tend to colonize the injured tissue, such as the pancreas, lung, liver, and intestine; (ii) CP-MSCs alleviate pancreatic injury and systemic inflammatory by inducing macrophage polarization from M1 to M2 in SAP rats; (iii) CP-MSCs secrete more TSG-6 in the inflammatory environment of SAP, thereby inducing macrophages to polarize from M1 to M2; (iv) TSG-6 secreted by CP-MSCs play a vital role in alleviating pancreatic injury and systemic inflammation in SAP rats. These findings provide a safe and effective therapeutic strategy for SAP and also provide new insights into the mechanisms responsible for the effectiveness of exogenous CP-MSCs.

Cell therapy is different from conventional drug therapy, mainly relying on seed cells to secrete a variety of cytokines and active molecules to exert a therapeutic role. Hence, the survival of CP-MSCs was crucial to exert better therapeutic efficacy in SAP. It is imperative to observe the survival status before investigating the therapeutic efficacy of CP-MSCs in SAP rats. The results of bioluminescence imaging revealed that the vast majority of exogenous CP-MSCs survived approximately for 72 h in the hyperinflammatory environment of SAP, which provided a reference for the selection of CP-MSC treatment endpoint.

Numerous studies have demonstrated that MSCs held the characteristics of migration and colonization to the injury site [32–34]. However, there is no consensus on whether exogenous MSCs colonize the pancreatic injury site in rodent animal models of SAP. Some studies believed that exogenous MSCs mainly resided in the lungs, and almost no MSCs colonized the pancreatic injury site [35]. Nevertheless, other studies suggested that MSCs could colonize the pancreas injury site, and also found that MSCs could differentiate into acinar-like cells [23, 36, 37]. In this study, we found that CP-MSCs partially colonized the lungs, and we also observed a large number of CP-MSCs in extrapulmonary organs, such as the liver, spleen, and intestine, etc. SAP is often accompanied by obvious intestinal and lung injury, while the heart and kidney generally have no obvious organic injury. We observed that plenty of CP-MSCs were colonized the pancreas, liver, duodenum, and colon of SAP rats, but only few CP-MSCs colonized the heart and kidney with abundant blood flow (Fig. S2). Furthermore, the number of CP-MSCs colonizing in the pancreas, liver, duodenum, and colon of SAP rats was significantly higher than that of control rats. Therefore, it showed that exogenous CP-MSCs owned the characteristics of colonization at the injured tissue of SAP. The colonization of CP-MSCs at the injury site will be more conducive to exert the therapeutic efficacy in SAP. Although, in rodent animal models of experimental SAP, MSCs tended to migrate and colonize the pancreas injury site, and some studies have confirmed that MSCs colonized in the pancreatic injury site could differentiate into acinar-like cells, the current mainstream view is that MSCs rely on the secretion of various cytokines or active molecules to play a protective role for SAP.

In the present study, one important discovery was that CP-MSCs alleviate pancreatic injury and systemic inflammation by inducing macrophages to polarize from M1 to M2 in SAP rats. Macrophages play a vital role in the progression from local inflammation of the pancreas to a systemic inflammation and multiple organ dysfunction, which makes macrophages an interesting therapeutic target for SAP. In this study, one interesting finding was that macrophages mainly showed M1 phenotype in the pancreas and liver tissues of SAP rats, and exogenous CP-MSCs could induce macrophage polarization from M1 to M2 phenotype. Another interesting finding was that when SAP rats were given simultaneously GdCl3 and CP-MSCs, the therapeutic efficacy of CP-MSCs was significantly enhanced, while when SAP rats were given simultaneously MCLs and CP-MSCs, the therapeutic efficacy of CP-MSCs was significantly weakened. This result might be explained by the fact that when GdCl3 and CP-MSCs were administered simultaneously in SAP rats, CP-MSCs promoted differentiation of monocytes towards anti-inflammatory macrophages and induced macrophage M2 polarization; meanwhile, GdCL3 depleted M1 macrophages by inducing apoptosis of M1 macrophages, so the proportion of M2 macrophages in the tissue obviously increased; when MCLs and CP-MSCs were administered simultaneously, CP-MSCs induced M2 polarization of macrophages; meantime, MCLs depleted M2 macrophages by inducing apoptosis of M2 macrophages, so the proportion of M2 macrophages in the tissues reduced significantly. M1 macrophages are pro-inflammatory macrophages, which secrete a vast array of pro-inflammatory factors (such as IL-1β, TNF-α, and iNOS) to aggravate the pancreatic and systemic inflammatory response, and M2 macrophages are anti-inflammatory macrophages, which secrete large amounts of anti-inflammatory factors (such as IL-10 and IL-4) to alleviate the pancreatic and systemic inflammatory response [27, 38]. Therefore, we can infer that CP-MSCs reduce pancreatic injury and systemic inflammatory response by inducing M2 polarization of macrophages in SAP rats.

Another important discovery was that CP-MSCs induced M2 polarization of macrophages by secreting TSG-6, and TSG-6 played a vital role in alleviating pancreatic injury and systemic inflammation in SAP rats. Tumor necrosis factor-α-induced gene/protein 6 (TSG-6) is an inflammation-inducing protein that can reduce tissue inflammation and promote damaged tissue repair in inflammatory diseases. For instance, Choi et al. found that human bone marrow-derived MSCs (BM-MSCs) could attenuate zymosan-induced mouse peritonitis by secreting TSG-6 to inhibit the production of pro-inflammatory factors of macrophages [39]. Qi et al. findings demonstrated that BM-MSCs accelerated wound healing and reduced tissue fibrosis by secreting TSG-6 in murine full-thickness skin wounds [40]. Song et al. revealed that BM-MSCs inhibited inflammatory neovascularization in the cornea by suppressing pro-angiogenic monocyte/macrophage recruitment in a TSG-6-dependent manner [41]. Meanwhile, some studies have shown that TSG-6 could induce the polarization of macrophages from a pro-inflammatory phenotype (M1) to an anti-inflammatory phenotype (M2). For instance, TSG-6 released from canine adipose tissue-derived (cAT)-MSCs could alleviate dextran sulfate sodium-induced colitis by inducing a macrophage phenotypic switch to M2 in mice [42]. Moreover, another study showed that TSG-6 secreted by human adipose tissue-derived (hAT)-MSCs induced macrophages that infiltrated into the colon to switch to the M2 phenotype, thus regulating the expression of inflammatory cytokines and the alleviation of DSS-induced colitis symptoms in mice [43]. Therefore, we speculated that CP-MSCs might regulate macrophage polarization by secreting TSG-6, thereby reducing pancreatic damage and systemic inflammation in SAP rats.

**Fig. 7:**
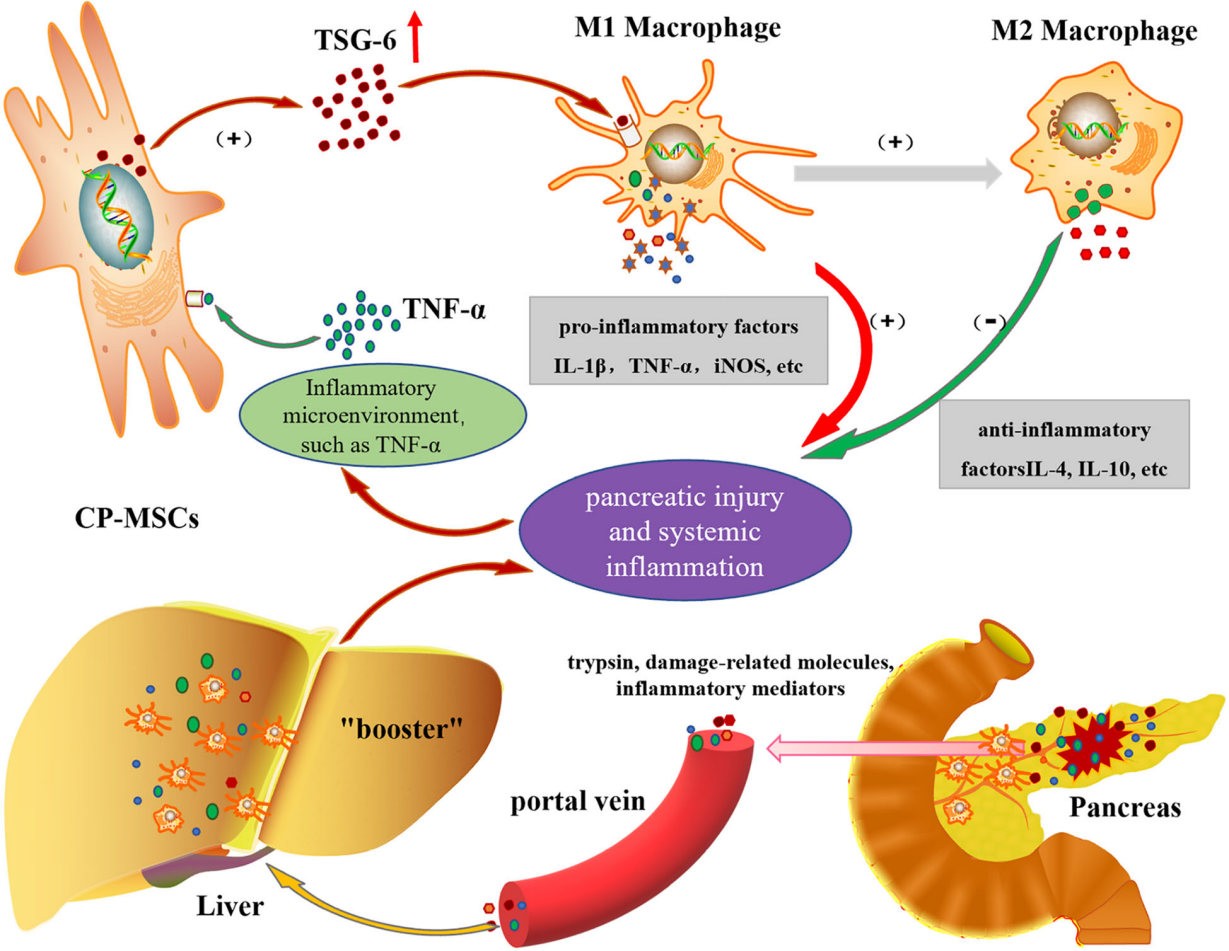
Schematic diagram depicting the potential therapeutic mechanism of CP-MSC to relieve SAP. During the occurrence of SAP, damaged pancreatic acinar cells release a variety of chemokines and inflammatory mediators to induce the recruitment of circulating monocytes to the pancreatic injury site and activate them into M1 macrophages, which in turn further aggravate pancreatic injury. When trypsin, damage-related molecules, and inflammatory mediators return to the liver through the portal vein, they can stimulate liver macrophages to secrete a large amount of pro-inflammatory factors, thereby further aggravating the systemic inflammatory. Therefore, liver macrophages play a “booster” role in promoting the development of local pancreatic injury to SIRS and multiple organ dysfunction. When CP-MSCs are exposed to the hyperinflammation environment of SAP, pro-inflammatory cytokines such as TNF-α stimulated CP-MSCs to secrete more TSG-6, TSG-6 regulated the polarization of macrophages from M1 to M2, and M2 macrophages secreted a large number of anti-inflammatory cytokines (such as IL-10 and IL-4) to inhibit excessive hyperinflammation and accelerate the repair of damaged pancreatic tissue

To confirm this hypothesis, we used RNA interference to knock down the expression of TSG-6 in CP-MSCs, and then co-cultured CP-MSCs (TSG-6 shRNA) with M1 macrophages in vitro. Meanwhile, CP-MSCs (TSG-6 shRNA) were transplanted into SAP rats to observe their treatment effects. When suppressing the expression of TSG-6 in CP-MSCs, we found that the ability of CP-MSCs to regulate the polarization of macrophages from M1 to M2 was significantly inhibited, and the protective effect of CP-MSCs on SAP rats was also significantly weakened. Therefore, it is shown that TSG-6 secreted by CP-MSCs exerted an important therapeutic role in SAP rats. Furthermore, we found that the inflammatory environment stimulated CP-MSCs to express TSG-6 higher. Meanwhile, studies have shown that when MSCs are placed in the inflammatory microenvironment, pro-inflammatory cytokines, such as TNF-α, stimulate MSCs to secrete TSG-6 [44, 45]. Taken together, we speculated that pro-inflammatory cytokines such as TNF-α stimulated CP-MSCs to secrete more TSG-6 in the inflammatory environment of SAP and TSG-6 regulated the polarization of macrophages from M1 to M2 (Fig. 7).

## Conclusion

In conclusion, our study provides a new treatment strategy for SAP and initially explains the potential protective mechanism of CP-MSCs on SAP rats. We found that CP-MSCs secreted TSG-6 to induce macrophages to polarize from M1 to M2, thereby reducing pancreatic injury and systemic inflammation in SAP rats. Despite the advancement in our understanding of the therapeutic effects of CP-MSCs in SAP, further study should be taken up using different animal models of SAP.

## Supplementary Information

**Additional file 1: **Experimental procedures. **Table S1.** Primer used for real-time quantitative PCR (RT-qPCR). **Figure S1.** CP-MSCs isolated from human placental chorionic plate MSCs meet the criteria of MSCs proposed by the ISCT. **Figure S2.** The distribution of CP-MSCs labeled with CM-Dil in the heart and kidney of rats with or without SAP. **Figure S3.** Isolation and identification of bone marrow macrophages.

## Data Availability

All data generated or analyzed during this study are included in this published article.

## Abbreviations

ANOVA: Analysis of variance
Arg-1: Arginase-1
BM-MSCs: Bone marrow-derived MSCs
CP-MSCs: Chorionic plate-derived MSCs
DMEM: Dulbecco’s modified Eagle medium
ELISA: Enzyme-linked immunosorbent assay
FBS: Fetal bovine serum
GdCl3: Gadolinium (III) chloride
IFN: Interferon
IL: Interleukin
iNOS: Inducible nitric oxide synthase
LPS: Lipopolysaccharide
MCLs: Mannosylated clodronate-encapsulated liposomes
M-CSF: Macrophage colony-stimulating factor
MPO: Myeloperoxidase
MSCs: Mesenchymal stem cells
P-MSCs: Placental-derived MSCs
SAP: Severe acute pancreatitis
SIRS: Systemic inflammatory response syndrome
TCA: Sodium taurocholate
TGF: Transforming growth factor
TNF-α: Tumor necrosis factor-α
TSG-6: Tumor necrosis factor-α-induced gene/protein 6
UC-MSCs: Umbilical cord-derived MSCs
