# Supplementary material for: Placental chorionic plate-derived mesenchymal stem cells ameliorate severe acute pancreatitis by regulating macrophage polarization via secreting TSG-6
Source: Stem Cell Res Ther. 2021 Jun 10;12:337. doi: 10.1186/s13287-021-02411-9 (PMC8193892; doi:10.1186/s13287-021-02411-9)
Supplement: Supplementary file 1 — Additional file 1: Experimental procedures. Table S1. Primer used for real-time quantitative PCR (RT-qPCR). Figure S1. CP-MSCs isolated from human placental chorionic plate MSCs meet the criteria of MSCs proposed by the ISCT. Figure S2. The distribution of CP-MSCs labeled with CM-Dil in the heart and kidney of rats with or without SAP. Figure S3. Isolation and identification of bone marrow macrophages. [file 13287_2021_2411_MOESM1_ESM.docx]

**Supplementary materials**

**Placental chorionic plate-derived mesenchymal stem cells ameliorate severe acute pancreatitis by regulating macrophages polarization via secreting TSG-6**

Qilin Huang^1,2#^, Xiumei Cheng^3#^, Chen Luo^4^, Shuxu Yang^2^, Shuai Li^1^, Bing Wang^1^, Xiaohui Yuan^1^, Yi Yang^1^, Yi Wen^1^, Ruohong Liu^1^, Lijun Tang^1*^, and Hongyu Sun^1,5*^

^1^Department of General Surgery & Pancreatic Injury and Repair Key Laboratory of Sichuan Province, The General Hospital of Western Theater Command, Chengdu 610083, China

^2^Tianjin Medical University, Tianjin 300070, China

^3^XinDu Hospital of Traditional Chinese Medicine & Chengdu 2nd Hospital of Traditional Chinese Medicine, Chengdu 610500, China

^4^Division of Hepatobiliary Pancreatic Surgery, Panzhihua Central Hospital, Panzhihua 617017, Sichuan Province, China

^5^Laboratory of Basic Medicine, The General Hospital of Western Theater Command, Chengdu 610031, China

# These authors contributed equally to this work

*Correspondence: Lijun Tang ([tanglj2016@163.com](mailto:tanglj2016@163.com)) or Hongyu Sun ( [shongyu2008@163.com](mailto:shongyu2008@163.com) )

**Experimental Procedures**

**Cell Counting Kit-8 (CCK-8) assay**

The proliferation of CP-MSCs was determined using the Cell Counting Kit-8 (CCK-8, Dojindo Molecular Technology, Japan). CP-MSCs were adjusted to a concentration of 2 × 10^4^ cells/ml with MSC Serum-Free Medium, and then 100 μl of it was added to each well of the 96well culture plate. After incubation for the first 24 h, the viable cell number was then tested every 24 h for seven consecutive days. Before the test, 10 μl CCK-8 reagent was added to each well, and the plates were incubated at 37 °C for 2 h. To determine the number of viable CP-MSCs, the optical density value at 450 nm was detected with a spectrophotometer (Multiskan GO, Thermo Scientific).

**Immunofluorescence Staining**

Immunofluorescence staining is used to detect the status of macrophage polarization in pancreas and liver tissues. Pancreas and liver samples were fixed in 10% buffered formaldehyde, embedded in paraffin, and sectioned. The 4 μm thick sections for antigen retrieval were permeabilized for 20 min with 0.5% Triton X-100. Subsequently, the slides were incubated with 5% BSA for 60 min at RT. The slides were stained by incubation with [Mouse anti-Rat CD68](https://www.citeab.com/antibodies/118739-mca341ga-mouse-anti-rat-cd68?des=2b2d0d5a48f5bef6) (Bio-Rad, USA), Rabbit anti Rat CD86 (Wuhan Servicebio Biotechnology Co., Ltd, China), Rabbit anti-Rat iNOS (Wuhan Servicebio Biotechnology Co., Ltd, China), Rabbit anti-Rat CD163 (Wuhan Servicebio Biotechnology Co., Ltd, China) and Rabbit anti-Rat Arg-1 (Wuhan Servicebio Biotechnology Co., Ltd, China) at the manufacturer’s recommended dilution at 4 ℃ overnight. Then, the slides incubated with CY3-labeled Goat Anti-Mouse and FITC-labeled Goat Anti-Rabbit secondary antibody (Boster Biological Engineering Co., Ltd., China) for 60 min at RT. Finally, the slides were stained with DAPI to visualize the nuclei. The distribution of the two phenotypes of macrophages in the pancreas and liver tissue was examined by laser scanning confocal microscopy.

**Flow cytometric analysis of macrophage apoptosis**

After the macrophages were prepared into a single cell suspension, the cell density was adjusted to 1 × 10^6^ cells/ml. Take 100 μl of cell suspension to EP tube, and then add 5 μl Annexin V-FITC and incubate at RT for 10 min in the dark. Subsequently, 5 μl of PI was added to each EP tube and incubated for 5 min at RT in the dark. Finally, 400 μl of PBS was added to the EP tube and the detection was completed within 1 h.

**Table S1. Primer used for real-time quantitative PCR (RT-qPCR)**

| mRNA | Forward sequence (5’-3’) | Reverse sequence (5’-3’) |
| --- | --- | --- |
| IL-1β (Rat) | CCCTGAACTCAACTGTGAAATAGCA | CCCAAGTCAAGGGCTTGGAA |
| TNF-α (Rat) | CGTCGTAGCAAACCACCAAG | CACAGAGCAATGACTCCAAAG |
| iNOS (Rat) | CAGCCCTCAGAGTACAACGAT | CAGCAGGCACACGCAATGAT |
| IL-10 (Rat) | AGAAGGACCAGCTGGACAACAT | CAAGTAACCCTTAAAGTCCTGCAGTA |
| IL-4 (Rat) | AACAAGGAACACCACGGAGA | ATTCACGGTGCAGCTTCTCA |
| Arg-1 (Rat) | ATCGGAGCGCCTTTCTCTAA | AGACCGTGGGTTCTTCACAA |
| CD163 (Rat) | CAACCGATGCTCAGGAAGAG | GATGGCACTTCCACATCCAA |
| GAPDH (Rat) | GTATGACTCTACCCACGGCAAGT | TTCCCGTTGATGACCAGCTT |
| TSG-6 (Human) | TTTCTCTTGCTATGGGAAGACAC | GAGCTTGTATTTGCCAGACCG |
| GAPDH (Human) | GGAGCGAGATCCCTCCAAAAT | GGCTGTTGTCATACTTCTCATGG |


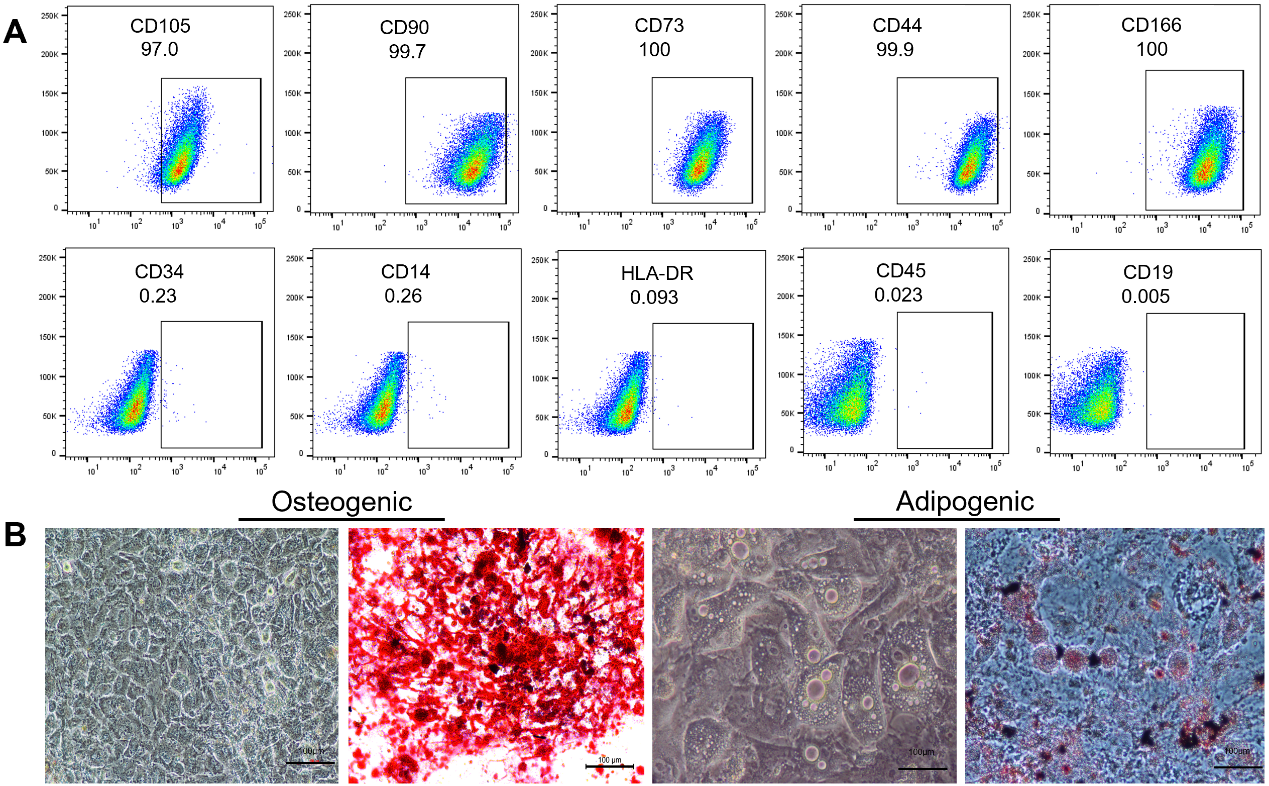


**Figure S1 CP-MSCs isolated from human placental chorionic plate MSCs meet the criteria of MSCs proposed by the ISCT**

(A) Cell surface markers of CP-MSCs were analyzed by flow cytometry. Immunophenotypic analysis of CP-MSCs exhibited a characteristic marker profile. CP-MSCs were positive for CD73, CD90, CD105, CD44, and CD166 (≥ 95%) and negative for CD45, CD34, CD14, CD19, and HLA-DR (≤ 2%). (B) The multilineage potential of CP-MSCs, osteogenic and adipogenic differentiation. Scale bars, 100 μm. *In vitro* differentiation assay showed that CP-MSCs were able to differentiate into osteogenic and adipogenic lineages**.** The above data indicated that CP-MSCs meet the criteria of MSCs proposed by the International Society for Cellular Therapies (ISCT).


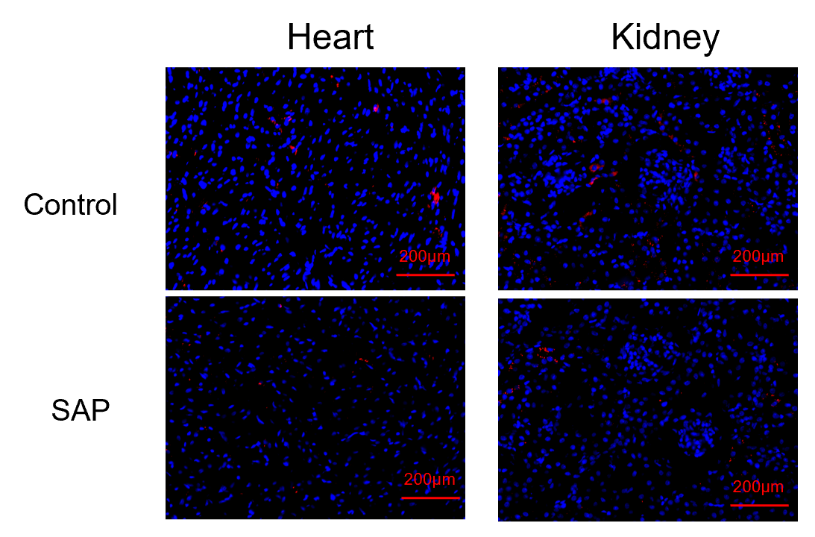


**Figure S2 The distribution of CP-MSCs labeled with CM-Dil in the heart and kidney of rats with or without SAP.**


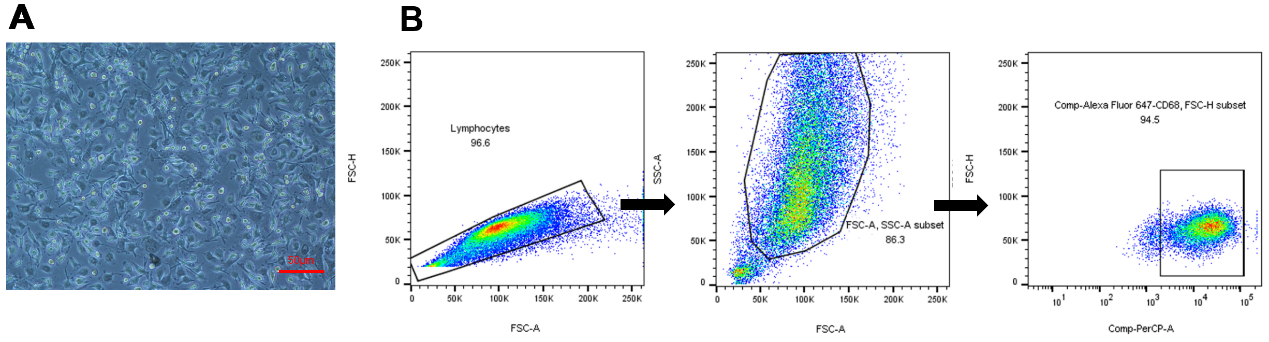


**Figure S3 Isolation and identification of bone marrow macrophages**

(A) Bone marrow-derived macrophages. Macrophages were isolated from rat bone marrow. Scale bars, 50 μm.

(B) Flow cytometric analysis of bone marrow macrophages, CD68+ cells are considered macrophages. Flow cytometry analysis showed that the purity of macrophages was about 90%.
